# The role of personal characteristics, work environment and context in working beyond retirement: a mixed-methods study

**DOI:** 10.1007/s00420-018-1387-3

**Published:** 2018-12-04

**Authors:** G. Lennart van der Zwaan, Karen M. Oude Hengel, Ranu Sewdas, Astrid de Wind, Romy Steenbeek, Allard J. van der Beek, Cécile R. L. Boot

**Affiliations:** 1 Department of Work Health Technology, Netherlands Organization for Applied Scientific Research TNO, Schipholweg 77-89, 2316 ZL Leiden, The Netherlands; 2 Body@Work, Research Center on Work, Health and Technology, TNO/VUmc, Amsterdam, The Netherlands; 3 Department of Public Health, Erasmus MC University Medical Center, Rotterdam, The Netherlands; 4 Department of Public and Occupational Health, Amsterdam Public Health research institute, VU University Medical Center, Van der Boechorststraat 7, 1081 BT Amsterdam, The Netherlands

**Keywords:** Bridge employment, Labor participation, Aging, Retirement, Longitudinal study

## Abstract

**Objective:**

To investigate the role of personal characteristics, work environment and context in working beyond retirement.

**Methods:**

In the current study, a mixed-methods design was applied including quantitative survey data and semi-structured telephone interviews. Respondents (*N* = 568) were selected from the Study on Transitions in Employment, Ability and Motivation (STREAM). Personal characteristics, work characteristics and contextual factors were measured using a questionnaire at baseline. Concurrently, qualitative data of 30 persons aged over 65 years were gathered. Logistic regression analyses were used to identify quantitative associations and thematic analyses were used for qualitative purposes.

**Results:**

Quantitative data revealed that being in good physical health (OR = 1.80), developmental proactivity (OR = 1.38), interesting work (OR = 2.02), appreciation (OR = 1.62) and voluntary work (OR = 1.58) were associated with working beyond the statutory retirement age. Additionally, qualitative findings suggested that working beyond retirement was mainly driven by the desire to contribute to society (e.g., mentor younger coworkers), and identified the employers’ willingness to hire an older worker despite existing stereotypes as an important precondition.

**Conclusions:**

Working beyond retirement is influenced by physical health and work characteristics, as well as motivational determinants such as the desire to contribute to society. However, to meet the increasing demands for paid jobs by individuals aged over 65 years, the willingness of employers to actually hire them is crucial. Therefore, recognition and utilization of older workers’ potentials is of great importance.

## Background

In the Netherlands, as in many other Western countries, the population is aging and this raises a pressure on public finances (Wheaton and Crimmins 2013; European Commission 2006). To counter this, governments stimulate workers by reforms of various institutions to work longer and delay retirement (Bloom et al. 2007; Staubli and Zweimüller 2013; Mastrobuoni 2009). For instance, they abolished early retirement opportunities and increased the statutory retirement age. As a result, the average retirement age in the Netherlands increased from 61 years in 2006 to 64 years and 5 months in 2016 (Centraal Bureau voor de Statistiek 2016; Centraal Bureau voor de Statistiek 2017c). Additionally, the net labor participation rate among persons aged 60–65 years increased from 24.9% in 2006 to 53.0% in 2016 (Centraal Bureau voor de Statistiek 2017a). Besides this larger proportion of workers working until the statutory retirement age, the proportion of persons who extend their working lives beyond the statutory retirement age increased as well. In the Netherlands, the total number of workers aged 65–75 years working beyond the statutory retirement age tripled from only 66 thousand in 2003 to 180 thousand in 2016 (Centraal Bureau voor de Statistiek 2017b). In 2016, the labor participation rate of Dutch individuals aged 65–75 years was 7.1%, which was slightly more than the 5.7% in the entire European Union (OECD 2016). These percentages remain relatively small compared to the 19.3% in the United States or the 22.8% in Japan (OECD 2016).

Working beyond retirement is also known as ‘bridge employment’, which refers to any form of paid employment after an individual retires and starts receiving a pension (Zhan et al. 2009; Wang et al. 2008). Bridge employment enables to earn extra income, but it also promotes psychological wellbeing, offers the opportunity to continue contributions to society and provides new opportunities to develop and improve skills and abilities (Wang and Shultz 2010; Deal 2007; Cahill et al. 2012). At the same time bridge employment may provide insight into how to maintain a (healthy) workforce with a large proportion of older workers, a major societal challenge in many Western countries (Topa et al. 2014; Foster and Walker 2013).

As bridge employment is a rather new phenomenon in recent years, emphasis has been placed on the reasons and preconditions of working beyond retirement. For example, recent insights suggested that good health—both physically and mentally—along with a need or desire to stay in employment are important reasons to work beyond the statutory retirement age (Demou et al. 2017). This was also seen in a study by de Wind et al. (2018) who found that workers without a chronic disease were more likely to work past the statutory retirement age compared to those with a chronic disease. Additionally, bridge employment offers the opportunity to engage in physical, cognitive and social activities which could lead to a higher quality of life. A study by Di Gessa et al. (2018) showed significantly lower wellbeing scores among those who are forced to stay in employment, because of financial issues, compared to those who continued work because they enjoyed their job.

To enable older workers to continue working past the statutory retirement age, an increasing amount of studies have been conducted on predictors of working beyond retirement. De Wind et al. (2016) studied a broad variety of potential predictors including individual characteristics, work motives and motivation, health, job characteristics, and the financial and social situation. They found that especially the motivation to work, physical health, the financial situation, and participation in voluntary work predicted working beyond retirement (de Wind et al. 2016). Additional research showed that working in healthcare, higher body height, and being intensively physically active predicted working past retirement (Scharn et al. 2017). Examples of work characteristics and social factors associated with working beyond retirement are low physical demands (Virtanen et al. 2017), flexible work arrangements (Pengcharoen and Shultz 2010), higher work ability, better work time control (Virtanen et al. 2014), a working spouse (Kim and Feldman 2000a), and children to support (Kim and Feldman 2000b). Veth et al. (Veth et al. 2018) focused on longitudinal relations between human resource management (HRM) and social support, and suggested that employers should mainly focus on creating high-quality relationships between bridge workers and their coworkers and supervisors rather than providing HRM bundles.

To date, only a few qualitative studies on the motives to work beyond retirement were conducted. For example, Sewdas et al. (2017) conducted interviews and focus groups and found that maintaining daily routines and the financial benefits were the most important motives. Reynolds et al. (2012) conducted 31 interviews and focused on outcomes rather than predictors and found that working beyond retirement increased financial security, health maintenance, and the continuation of personal development.

In sum, working beyond retirement is driven by multiple factors in the domains of personal characteristics, work characteristics and contextual factors. However, the majority of previous studies relies on quantitative data or qualitative data solely, whereas a mixed-methods design could provide important implications for this relatively new phenomenon based on cross-validation of findings. The current study further builds on the study by de Wind et al. (2016). As bridge employment has become more prevalent in recent years, adding an extra follow-up measurement and applying an etiologic model instead of a prediction model will provide additional knowledge on determinants of bridge employment. Therefore, the aim of the current study is to gain insight in the etiology of working beyond retirement. In line with this aim, this study addresses the following research questions: (1) What is the influence of personal characteristics, work characteristics and contextual factors on working beyond the statutory retirement age? (2) In what ways do personal characteristics, work characteristics and contextual factors differ for those working beyond retirement and those who do not?

## Methods

### Study design

In the current study, a convergent parallel mixed-methods design was applied including semi-structured telephone interviews and quantitative survey data (Creswell 2012). First data were collected and analyzed concurrently. Thereafter, both quantitative and qualitative results were merged into the three different content areas (i.e., personal characteristics, work characteristics, contextual factors) which allowed for comparison.

This study utilized data from the longitudinal cohort Study on Transitions in Employment, Ability and Motivation (STREAM). Persons aged 45–64 years participated in the GFK-Intomart online panel and filled out an online survey in 2010 (T1), 2011 (T2), 2012 (T3), 2013 (T4), and 2015 (T5). In total, 15,118 individuals (employees *N* = 12,055, self-employed persons *N* = 1029, and persons without paid employment *N* = 2034) participated in the STREAM baseline measurement. The STREAM survey contained questions on the following topics: health, job characteristics, skills and knowledge, social factors, financial factors, ability, motivation, opportunity, productivity, and transitions in employment. The Medical Ethical Committee of the VU Medical Center Amsterdam declared that the Medical Research Involving Human Subjects Act (abbreviation in Dutch: WMO) did not apply to STREAM. Information accompanying the online questionnaire emphasized that privacy was secured and data were stored in secured computer systems. Detailed information on STREAM can be found elsewhere (Ybema et al. 2014).

### Study sample

**Fig. 1:**
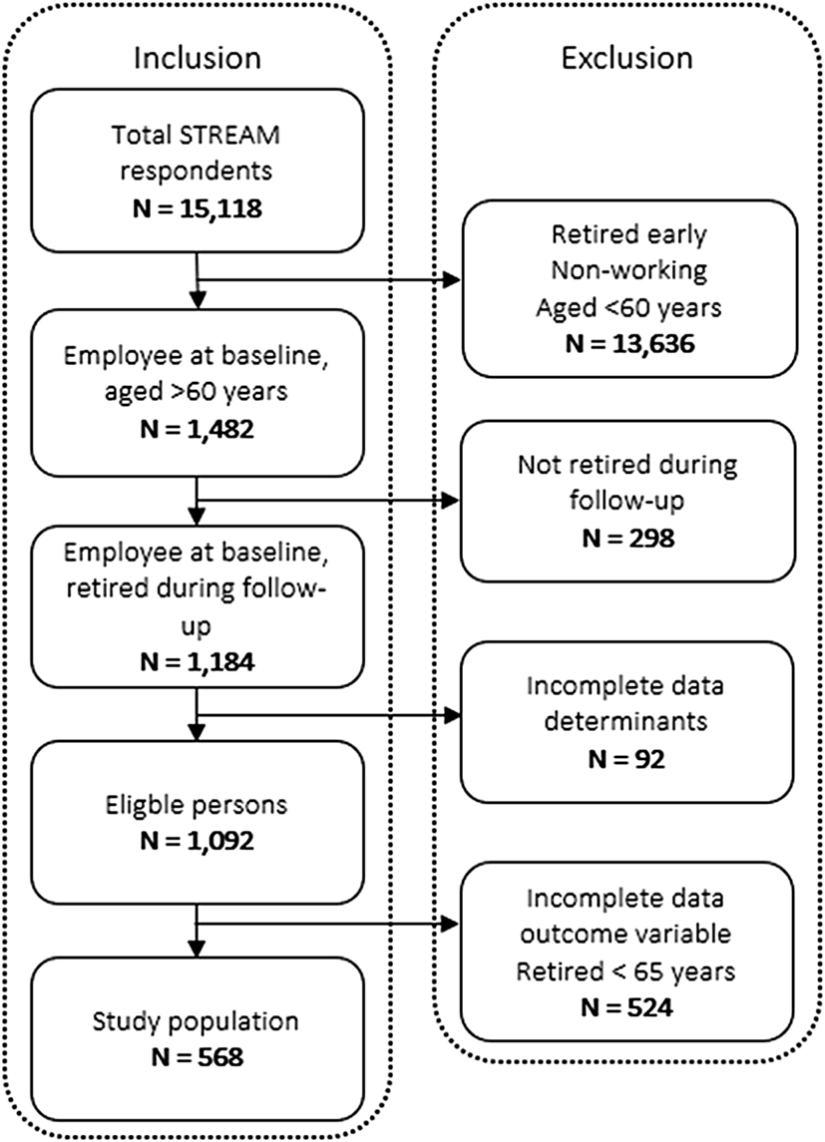
Flow of the study population resulting in 568 persons included in the current study

For the purpose of the current study, all STREAM respondents who were employee at baseline (T1), who reached the statutory retirement age (i.e., 65 years) during follow-up (T1–T5), and who had given permission for additional research were selected (*N* = 1482). All persons who did not retire during follow-up were excluded (*N* = 298). Respondents with missing data on either the determinants or the outcome variable were also excluded (*N* = 616). Figure 1 presents a flow chart of the study population (*N* = 568).

### Quantitative phase

Based on an earlier study by de Wind et al. (2016), three broad content areas were formed, namely personal characteristics, work characteristics, and contextual factors. All variables were derived from the STREAM questionnaire.

The included independent variables were retrieved from the baseline measurement. The timing of the outcome variable depended on the year a person worked as an employee or self-employed person, reached the statutory retirement age (i.e., 65 years) and indicated that he/she received an old-age pension. This follow-up measurement could occur at T2, T3, T4 or T5. In the analysis those who were retired, but also still working were compared to those who were retired and did not work anymore.

## Dependent variable

### Working beyond retirement

Working beyond retirement was assessed with a single item on employment status “what situation are you currently in?” based on the Netherlands Working Conditions survey (NWCS) (Koppes et al. 2010). Respondents chose one or more of the following answer categories: one paid job as an employee; multiple jobs as an employee; self-employed; unemployed; disability pension; (early) retirement; attending school; housemen/wife. Working beyond retirement was defined as having a paid job as an employee or self-employed person, while receiving an old-age pension on one of the four follow-up measurements. For purposes of the current study persons who worked while receiving an old-age pension were compared to those receiving an old-age pension while being fully retired.

The Dutch pension system incorporates different models of pension funding [i.e., (1) old-age pension, (2) supplementary pension schemes, and (3) personal savings]. Between 2012 and 2021, the statutory retirement age is being raised from 65 to 67 years. In the current study, retirement referred to withdrawal from paid work among persons aged over 65 years.

## Independent variables

### Personal characteristics

Personal characteristics included self-perceived mental and physical health, mastery, work motives, work engagement, and developmental proactivity. Prior to the analysis, for the current study population, Cronbach’s alphas were calculated to assess reliability of the independent variables consisting of two or more items.

Self-perceived physical and mental health were assessed using the Short Form-12 Health Survey (Ware et al. 1996). Physical and Mental Health Composite Sores were computed using the scores on the 12 items. All scores range from 0 to 100, with higher scores reflecting better health. The physical health score includes items such as ‘Thinking about the past 4 weeks, have you accomplished less than you would like as a result of your physical health’. The mental health score includes items such as ‘During the last 4 weeks, did you have trouble doing work or other activities as carefully as usual as a result of an emotional problem, such as feeling depressed or anxious?’ Given the skewed distributions the 25th and 75th percentile were used to discriminate between poor, moderate and good physical health as well as mental health.

Mastery was assessed using a 7-item scale derived from the Pearlin Mastery Scale (Cronbach’s alpha 0.81) (Pearlin et al. 1981). Mastery is considered a physiological resource reflecting the extent to which individuals experience control on factors influencing their life situation. Answers were given on a 5-point scale (“strongly disagree” to “strongly agree”) and higher scores reflect higher degrees of mastery. Mastery was analyzed as a continuous variable.

Work motives included in this study were self-constructed and based on the Self Determination Theory (Ryan and Deci 2000). Based on four questions participants indicated the extent to which they enjoyed working, found work meaningful, worked for financial reasons, and worked because others expect them to do so. Working because someone enjoys working was measured with three-items (Cronbach’s alpha 0.72): maintaining daily routines, social contacts, and enjoyment of work. Working because someone finds work meaningful was measured with two items (Cronbach’s alpha 0.81): the desire to contribute to society and the extent to which work contributes to the meaning to life. Working for financial reasons was measured with one item: working to earn money. Working because others expect them to do so was also measured with one item: working because relatives and friends think it is important. Answers were given on a 5-point scale (“totally disagree” to “totally agree”). Given skewed distributions the 75th percentile was used to discriminate between low and high degrees of one of the four work motives.

Work engagement was assessed using six-items on vigor and dedication of the Utrecht Work Engagement Scale (UWES) (Schaufeli et al. 2006). Vigor refers to the degree of physical or mental strength, and dedication refers to the willingness to devote time and energy to something important. Answers were given on a 7-point scale (“never” to “always”) and higher scores reflect a higher work engagement. Both dimensions were combined to one scale (Cronbach’s alpha 0.93) and analyzed as a continuous variable.

Developmental proactivity was measured using a 4-item scale derived from Van Veldhoven and Dorenbosch (Van Veldhoven and Dorenbosch 2008). Respondents were asked (1) to which extent they actively seek for opportunities in their job to develop their knowledge and skills, and (2) to what extent they prepare for future changes in their jobs. Answers were given on a 5-point scale (‘strongly disagree’ to ‘strongly agree’, Cronbach’s alpha 0.81). Higher scores reflect a higher desire to further skills and knowledge. Developmental proactivity was analyzed as a continuous variable.

### Work characteristics

Work characteristics included in this study were physical demands, job demands, autonomy, social support, appreciation, and the degree to which work was interesting.

Physical demands were assessed using a 5-item scale on uncomfortable postures, force exertion, the use of vibrating tools, prolonged standing and prolonged squatting, based on the Dutch Musculoskeletal Questionnaire (Cronbach’s alpha 0.83) (Bot et al. 2004). Answers were given on a 3-point scale (“yes, regularly”, “yes, sometimes”, and “no”). Given skewed distributions the 25th and 75th percentile were used to discriminate between low, moderate and high physical demands.

Job demands were assessed using a 4-item scale from the Job Content Questionnaire (JCQ, Cronbach’s alpha 0.89) (Karasek et al. 1998). Answers were given on a four-point scale (“always” to “never”) and higher scores reflect higher job demands. Job demands was analyzed as a continuous scale.

Autonomy was assessed using a five-item scale from the JCQ (Cronbach’s alpha 0.79) (Karasek et al. 1998). Answers were given on a three-point scale (“yes, regularly”, “yes, sometimes” and “no, never”) and lower scores reflect a lower degree of job autonomy. Autonomy was analyzed as a continuous scale.

Social support was defined as the degree to which colleagues and supervisors are willing to help and listen to work-related problems. Support was measured using four items from the Copenhagen Psychosocial Questionnaire (COPSOQ, Cronbach’s alpha 0.81) (Kristensen et al. 2005). Answers were given on a 5-point scale (“always” to “almost never”) and lower scores reflect a lower degree of social support. Social support was analyzed as a continuous variable.

Furthermore, based on a question derived from the NWCS (Koppes et al. 2010), employees were asked to indicate the extent to which the following aspects are present at work: appreciation, interesting work, and opportunities for learning and development. Answers were given on a 4-point scale (“not present at all” to “highly present”) and lower scores reflect a lower appreciation and lower interesting work. Answer categories were dichotomized into not present (“not present at all” and “somewhat present”) and present (“rather present” and “highly present”).

### Contextual factors

Contextual factors included the financial situation of the household, participation in voluntary work, informal care provision and the employment status of the partner.

The financial situation was measured by a single item “What is the financial situation of your current household?” derived from the OSA panel 2008 (Van Echtelt et al. 2016). Answers were given on a 5-point scale (“very short of money” to “a lot of money left”). Lower scores reflect a poor financial situation. Answers were categorized into money left (“some money left” and “a lot of money left”), just adequate (“just adequate”) and short of money (“very short of money” and “somewhat short of money”).

Voluntary work was assessed using a self-constructed single item “Have you spent time on voluntary or charity work during the last 12 months?” based on the OSA panel 2008 (Van Echtelt et al. 2016). Answers were given on a dichotomous scale (“yes” “no”). Informal care was also assessed using a single item “Have you spent time on providing informal care over the past 12 months” based on the OSA panel 2008 (Van Echtelt et al. 2016). Answers were given on a dichotomous scale (“yes” or “no”). Employment status of the partner was assessed using a single item “In what situation is your partner currently?” using the NWCS (Koppes et al. 2010). Answers were given on an 8-point scale and categorized into not working (“unemployed”, “unfit for work”, “housewife/houseman”, “retired”, “attending school”, and “involved in voluntary work or informal caregiving”), working (“employed” and “self-employed”), and no partner.

### Covariates

Individual factors such as age, gender and educational level were included as potential confounders. The highest level of education was coded according to the 1997 International Standard Classification of Education (ISCED-97) and categorized into three groups: low (primary school, lower and intermediate secondary school, or lower vocational training), intermediate (higher secondary school, or intermediate vocational training), and high (higher vocational education, university education). Age was added on a continuous scale.

### Statistical analyses

The main interest in this study was the longitudinal association between independent determinants and working beyond retirement. Therefore, a specific model was constructed for each determinant in three steps. First, crude odds ratios were calculated using univariate logistic regression models between each determinant and working beyond retirement. In the second step, each potential confounder (i.e., all independent variables, including covariates, listed above) was added to the crude models for each independent determinant separately. The potential confounders were added using a stepwise forward procedure. The strongest confounders were retained based on the largest change in regression coefficient of the determinant. Again, in the final third step, all remaining confounders were added step by step for each independent determinant separately and the extent to which adding these variables changed the odd ratios was assessed. A 10% change in the odds ratio was considered as relevant to justify adjustment (Twisk 2007; Grobbee and Hoes 2009). These analyses were carried out using SPSS version 24.0.

## Qualitative phase

To ensure comprehensive reporting of qualitative findings, the consolidated criteria for reporting qualitative research (COREQ) (Tong et al. 2007) were taken into account. The COREQ criteria, a 32-item checklist, include aspects of the research team, methods, the context of the study, results, analysis and interpretations. A team of academic researchers conducted this qualitative phase: LvdZ, RaS under supervision of CB and RS.

### Participant selection

Interview participants were recruited from the original STREAM study population. Participants aged over 65 years were eligible for the interview study if they participated in the fifth wave of data collection in 2015, and had given permission to be contacted for additional research. In total, 281 persons were eligible for inclusion.

Two contrasting groups (e.g., those working beyond retirement versus those fully retired) of interviewees were selected by sampling on employment status. By selecting these two groups, the current study is able to gain insight into both barriers and facilitating factors of working beyond retirement. Additionally, we purposefully sampled on age, gender, educational level, and health status. This is also known as maximum variation sampling (Patton 2015). Selection on age took place since different motives might underlie working beyond retirement immediately after reaching the statutory retirement age compared to re-entering the labor market at the age of, for example, 67 years. Selection on educational level took place because working beyond retirement might depend on different exposures (determined by educational level) in preretirement jobs. The same reasoning applies for gender and health status. Between January and February 2016, 61 participants were invited by telephone (by LvdZ): after explaining the purpose of the study, consent was documented and an appointment planned. Reasons for not taking part were: not responding to our call (*N* = 18), being sick (*N* = 1) or having difficulties with speaking due to a stroke (*N* = 1). Another 11 individuals were not eligible. The recruitment process stopped after data saturation was reached, meaning that no new insights were obtained in the last few interviews. Therewith, a total of 30 individuals participated in the qualitative phase.

### Interview procedure

Interviews were conducted by a male and a female researcher, LvdZ and RaS, from February to May of 2016. Prior to the data collection, a semi-structured interview guide (available upon request) based on the following topics was created: (1) reasons for working beyond the retirement age; (2) considerations about leaving work; (3) the timing at which people decide to remain active or retire; (4) persons who played a role in their decision; (5) planning for the future (e.g., retirement). See Table 4 in Appendix A for the full topic list. The two interviewers conducted three pilot interviews to adjust and optimize the protocol. Before starting the interview, the interviewer introduced him/herself the research objectives and informed the participant about anonymity and confidentiality. Interviews lasted between 30 and 60 min. During the interviews, the protocol was used to take detailed notes and quotes from the participants’ responses. Afterwards these notes and quotes were saved.

### Analyses

Data analysis was an ongoing process in which coding the earlier interviews was alternated with conducting additional interviews. This allowed for further exploration of existing themes—which was stimulated by including a broad variety of participants through purposefully sampling—and monitoring of data saturation. To ensure decent comparability, all participants were interviewed with the same topic list. Towards the last interviews no new information was found.

Data were analyzed based on thematic coding. Notes of the first three interviews were read and re-read to become immersed with the content. Aspects relevant for answering the research question were open-coded and cross-checked by two independent researchers (LvdZ and RaS). Next, the codes were extensively discussed by LvdZ and RaS, differences in interpretation were resolved by consensus. In the second phase, the remaining 27 persons were interviewed, codes were clustered and thematically grouped following the classification of the quantitative analyses: personal characteristics, work characteristics, and contextual factors. To increase reliability of the analysis, the themes and categories emerged were discussed within the project group until consensus was achieved.

## Results

Descriptive information on the study population (*N* = 568) is presented in Table 1. In total, 28.3% of the participants worked beyond the statutory retirement age (*N* = 161). The group of persons working beyond retirement was slightly older at baseline (62.9 years versus 62.2 years), consisted of more men (55.3% versus 49.1%), were higher educated (33.5% versus 30.7%), and indicated they liked to work more often (84.5% versus 38.6%) when compared to those not working beyond retirement.

**Table 1:** Quantitative phase: baseline characteristics of the study population (*N* = 568)

	Working beyond retirement (*N* = 161)			Not working beyond retirement (*N* = 407)		
	%	Mean	IQR^a^	%	Mean	IQR^a^
Personal characteristics						
Age						
60–64		62.9	62.0–64.0		62.2	61.0–63.0
Gender						
Male	55.3			49.1		
Education						
Low	31.1			33.2		
Inter	35.4			36.1		
High	33.5			30.7		
Physical health						
Poor	24.8			24.6		
Moderate	47.2			52.6		
Good	28.0			22.9		
Mental health						
Poor	23.6			24.8		
Moderate	50.3			45.5		
Good	26.1			29.7		
Mastery						
1–5		3.9	3.4–4.1		3.8	3.4–4.1
Working because someone likes to work						
Yes	84.5			38.6		
Working because it is meaningful						
Yes	35.4			32.9		
Working for financial reasons						
Yes	35.4			39.8		
Working because of expectations of others						
Yes	18.6			23.6		
High work engagement						
Yes		4.9	4.3–5.7		4.6	4.0-5.7
Developmental proactivity						
1–5		3.9	3.5–4.3		3.8	3.5-4.0
Work characteristics						
Physical demands						
High	23.6			20.1		
Medium	29.2			38.6		
Low	47.2			41.3		
Job demands						
1–5		2.8	2.3–3.5		2.9	2.5–3.5
Autonomy						
1–5		3.9	3.5–4.6		3.9	3.6–4.4
Social support at work						
1–5		3.5	3.0–4.0		3.5	3.0–4.0
Appreciation						
Present	65.8			59.0		
Interesting work						
Present	34.8			22.6		
Opportunities for learning and development						
Present	8.7			8.1		
Contextual factors						
Employment status partner						
Not working	37.9			36.4		
Working	32.3			30.7		
No partner	29.8			32.9		
Informal care						
Yes	16.1			16.0		
Voluntary work						
Yes	44.7			36.4		
Financial situation of the household						
Money left	64.6			60.0		
Adequate	19.9			24.3		
Deficit	15.5			15.7		

^a^Interquartile range (25th–75th percentile)

Interviews were conducted with 30 participants; 15 persons who worked beyond the statutory retirement age and 15 who were fully retired; 14 males and 16 females; the mean age of the study population was 66 years. Most respondents (*N* = 22) reported a good self-perceived health status. Characteristics are shown in Table 2.

**Table 2:** Qualitative phase: characteristics of interview participants (*N* = 30)

	Working beyond retirement (*N* = 15)	Fully retired (*N* = 15)
Age (years)		
Range	65–69	65–69
Gender		
Male	7	7
Female	8	8
Educational level		
Low	3	5
Intermediate	5	6
High	7	4
Employment status		
Employed	7	N/A
Self-employed	8	N/A
Self-perceived health status		
Poor	2	6
Good	13	9

*N/A* not applicable

### Personal characteristics

The adjusted model—see Table 3—showed that participants with good physical health were more likely (OR = 1.80; 95% CI 1.02–3.17) to work beyond the statutory retirement age compared to participants with poor physical health. Good mental health was significantly associated with working beyond retirement in the crude analyses, but was not statistically significant in the adjusted model. In addition, developmental proactivity was significantly associated with working beyond retirement (OR = 1.38; CI 1.01–1.89).

**Table 3:** Quantitative phase: Longitudinal associations between personal characteristics, organizational/work characteristics and contextual factors, and working beyond retirement

	Crude analyses		Adjusted analyses	
	OR	95% CI	OR	95% CI
*Personal characteristics*				
Physical health				
Low	1.00	Ref	1.00	Ref
Moderate	1.31	0.82–2.09	1.34^a^	0.81–2.20
Good	1.83	1.09–3.07	1.80^a^	1.02–3.17
Mental health				
Low	1.00	Ref		
Moderate	1.50	0.93–2.42		
Good	2.01	1.18–3.41		
Mastery	1.30	0.96–1.75		
High work engagement	1.26	1.06–1.49		
Developmental proactivity	1.24	0.91–1.67	1.38^b^	1.01–1.89
Educational level				
Low	1.00	Ref		
Intermediate	1.05	0.67–1.64		
High	1.17	0.74–1.84		
Work characteristics				
Job demands	0.84	0.68–1.03		
Interesting work	1.83	1.23–2.72	2.02^c^	1.34–3.04
Appreciation	1.34	0.92–1.96	1.62^c^	1.07–2.45
Social support at work	0.83	0.67–1.02		
Physical demands				
High	1.00	Ref		
Medium	1.30	0.78–2.16		
Low	1.49	0.92–2.42		
Contextual factors				
Voluntary work	1.42	0.98–2.05	1.58^b^	1.07–2.33

*Ref* reference category

^a^Adjusted for age and work engagement

^b^Adjusted for age

^c^Adjusted for social support

These findings were supported by the individual interviews, in which health status appeared to be an essential precondition in the decision-making process. Both employees who worked beyond retirement and retirees who fully retired stressed the influence of a good physical and mental health condition on the decision-making process. For example, poor physical health forced few participants to retire (early). Participant A (female, retired) explained: “Due to the osteoarthritis and migraine I was forced to stop working. I wanted to continue but my general practitioner advised me to apply for a less physically demanding job. Because I only had vocational training and I was 62 at that time I could not find a new job and retired”. Next, participants stressed the positive effects of working beyond retirement on both physical and mental health. Participant B (male, employed, professional services) mentioned: “Because I felt fit by the time I retired I decided to prolong my career. I simply want to work, it keeps me going and I feel fit”. On the contrary, once confronted with the finiteness of life, many individuals decided to fully retire and enjoy the preferences of retirement while being in good health. Participant C (male, retired) said: “No, take my father for example who had a 50-year career and died shortly after. We need to ensure as much time as possible to enjoy the good things in life”.

In addition to health, several interviewed respondents (20%) mentioned they were working beyond retirement as they would like to transfer skills and share knowledge with younger coworkers. Participant D (male, self-employed, financial services) explained: “In the near future I would like to have some kind of mentoring role. I’d like to prepare someone to take over my job”.

### Work characteristics

Based on the quantitative data, participants who experienced their work as being interesting were more likely (OR = 2.02; CI 1.34–3.04) to work beyond retirement, and the same was found for participants who felt appreciated by colleagues and supervisors (OR = 1.62; CI 1.07–2.45; Table 3). The latter was also found in the interviews in which respondents indicated they continued their career to complete a project their employer asked them to. Contrary to what was expected none of the work motives was associated with working beyond retirement. This was also found for educational level.

One of the primary themes according to the interviews was access to work beyond retirement provided by the employer. Some participants mentioned that having an employer who allowed to work beyond retirement was crucial in their decision to do so. On the contrary, there were also some negative experiences with employers declining the opportunity to hire an older worker despite his or her specific knowledge and experience. Participant E (female, retired) applied for the same job at a different company and explained: “I enjoyed my work a lot, I even offered to continue on a self-employed basis but there was no chance that they allowed me—being 65 at that time—to stay”.

Additionally, work offers the opportunity to maintain contact with clients and colleagues, and to avoid the so-called black hole of retirement. Most interview respondents mentioned these were important motives to work beyond retirement. For example, participant F (female, self-employed, commerce) explained: “Over the past years I’ve build up a rather large clientele with whom I want to stay in touch. They expect me to continue and I prefer to do so, this is very much appreciated”.

Another mentioned factor influencing the decision-making process was the degree of flexibility that the job offers. Respondents mentioned they desired the opportunity to trade-off between work and leisure. Sixty-year-olds are taking care (socially and financially) of their parents, children and/or grandchildren. Therefore, they are looking for contracts with a flexible number of working hours allowing them to combine informal care duties, taking care of grandchildren, and work. Participant G (female, self-employed, health services) said: “I combine work with caring for my grandchildren. Since I am self-employed I manage my own hours and decisions. I’m no longer forced to work but I just want to”.

### Contextual factors

Participants who are involved in voluntary work were more likely (OR = 1.58; CI 1.07–2.33) to work beyond the statutory retirement age (Table 3).

Based on the interviews, other contextual factors including the financial situation influenced the decision-making process. The financial situation resulted in employment beyond retirement through roughly two different pathways.

First, a number of respondents worked beyond retirement because they had to compensate for financial shortcomings. For example, participant H (female, employed, sector undisclosed) explained: “The pension my husband had in Israel was invalid here. Now I receive an old-age pension and although I’m very grateful this little amount of money is not enough to make ends meet, therefore I’m forced to stay at work”. Second, the extra income provided the opportunity to undertake leisure activities or to supplement savings. For example, participant I (male, employed, sector undisclosed) explained: “I’m not forced to work to pay off a mortgage or whatsoever, but I want to work because it offers me the financial opportunity to travel”.

Another factor influencing the decision to work beyond retirement was the employment status of the partner. Those with a working partner were more inclined to work beyond retirement. Participant J (male, employed, sector undisclosed) said: “My wife has to continue for four more years so I decided to continue. I don’t want to be home alone”. In addition, some respondents mentioned they continued to work because they have certain worries about life as a retiree. Participant K (female, self-employed, sector undisclosed) stated: “I need something to do, work provides a purpose to get up and contribute to society”.

## Discussion

Both quantitative and qualitative data showed that being in good-physical-health was associated with working beyond the statutory retirement age. Alongside a good health condition, quantitative data showed that developmental proactivity, interesting work characteristics, appreciation by colleagues and supervisors, and voluntary work increased the likelihood of working beyond retirement. According to the qualitative results, respondents worked beyond retirement because it gave them a reason to get up in the morning, it fulfilled their desire to contribute to society and it offered them the opportunity to maintain social contacts. Another important precondition originating from the interviews is the willingness and attitudes of employers towards hiring workers aged over 65 years. The influence of work motives remained unclear, no association was found in the quantitative phase, while several interviewees mentioned they worked because it fulfilled their desire to contribute to society, which is similar to the motive “working because it is meaningful”. It could well be that this desire to contribute to society is fulfilled through other activities such as voluntary work or informal care provision.

### Personal characteristics

As expected, good-physical-health enabled older workers to work beyond the statutory retirement age. This finding supports previous research showing that good health—both physically and mentally—(Demou et al. 2017) and the absence of a chronic disease (De Wind et al. 2018) are associated with working beyond retirement. A good health condition is also an important factor in the decision-making process to retire or work beyond the retirement age (Dingemans et al. 2016). This is also called the health selection mechanism, in which people with poor health are more likely to exit the work force via disability schemes, early retirement or economic inactivity before reaching the statutory retirement age (Claussen et al. 1993; McMichael 1976).

Additionally, developmental proactivity was associated with working beyond the statutory retirement age. Qualitative findings from the current study complemented this by showing that participants are seeking for jobs in which they have the ability to keep learning. This finding contradicts the general perceived perception on older workers’ restraint willingness to invest in their own knowledge and skills, which may even result in age discrimination at work through fewer opportunities for training and development (Posthuma and Guerrero 2013; Feyrer 2008; Fasbender 2016). Diverse reasons could explain this contradiction, such as differences by educational level in willingness to engage in educational activities during working life (Fouarge et al. 2013), and those in lower socioeconomic positions feeling uncertain about their learning abilities (Illeris 2006). Within the current study, approximately 75% of the persons working beyond retirement had a moderate to high educational level which could explain the association found.

### Work characteristics

Based on the quantitative findings interesting work and appreciation are the most important factors—within this domain—encouraging older workers to work beyond retirement. This appreciation aspect is in line with the study by Veth et al. (2018) which highlighted the importance of creating high-quality relationships at the workplace. Contrary to what was expected work motives were not associated with working beyond retirement. However, qualitative findings, suggested that those working beyond retirement desire to (1) contribute to society, and (2) tasks allowing them to mentor their younger counterparts. These findings support previous studies that found that older workers are motivated by different aspects of their jobs compared to younger workers, especially if the job foresees in the possibility to perform fulfilling work including mentoring tasks (Boumans et al. 2012; Finkelstein et al. 2003; Doerwald et al. 2015). This strong desire could originate from the so-called fear of missing out, especially when the contribution to society is no longer anchored to employment.

Another important precondition—based on the qualitative data—is the extent to which an employer offers opportunities and conditions for career extension. Unfortunately, this aspect was not present in the quantitative phase. However, previous research on work-related factors predicting the retirement decision-making process showed that a supporting and social climate could reduce the attractiveness of early retirement (Van Solinge and Henkens 2014). However, the employers’ willingness to hire older workers is limited (Van Dalen et al. 2009). Among others, this is due to the assumption that the return on investment ratio—wage versus productivity—might be less among older workers compared to younger workers (Ostroff and Atwater 2003; Fouarge and Montizaan 2015). Although most stereotypes regarding older workers are negative (i.e., resistance to change, poor learning abilities, and an overall declining job performance), also many positive stereotypes (e.g., being more dependable, loyalty, and higher levels of interpersonal and organizational trust) exist (Dordoni and Argentero 2015). In other words, empirical evidence is inconclusive. In an attempt to encourage employers to rethink their attitudes towards older workers, it is important to note that many of these stereotypes are based on preconceived ideas or unfounded assumptions as was probably the case within the interviews in the current study.

### Contextual factors

Regarding contextual factors the quantitative data revealed that participation in voluntary work was associated with working beyond retirement. Respondents indicated that voluntary work provided the opportunity to contribute to society and maintain social contacts. Moreover, voluntary work could be seen as an approach to counter adverse health effects accompanying retirement, such as decreased cognitive capacities (Nexo and Borg 2016; Teng et al. 2012; Müller et al. 2015) as it offers the opportunity to develop new knowledge and skills (Davila and Diaz-Morales 2009). It could be hypothesized that working beyond retirement counter similar factors as participation in voluntary work. The main difference between both types of social participation is the degree of voluntariness which highlights the importance of the financial situation. Regarding this financial situation, it is expected that in the near future more people are forced to continue to work because of insufficient pension savings, especially among those in low socioeconomic positions (OECD 2014; Lusardi and Mitchel 2007). This was also found in a previous quantitative study by de Wind et al. (2016) who showed that those in a poor financial situation are more likely to work beyond retirement compared to those in better financial situations. Contrary, this association was not found in the current study which can be explained by the relatively high educational level in the study population.

### Strengths and limitations

The strength of the current study is the mixed-methods approach that allowed for cross-validation of quantitative findings. For example, this approach allowed for further exploration of work motives, which proved to be very relevant in the qualitative phase but was non-significant in the quantitative phase. Other strengths are the saturation of qualitative findings that had been achieved, and the large study sample which allowed to incorporate a relatively high number of independent variables in the quantitative phase. However, some limitations should be considered as well. First, selection bias might occur due to the selection of the study population. The high number of missing cases occurred (42%) due to missing data on either determinants or outcome variable. This might have led to an underestimation of the associations found, and should be considered when interpreting the results found. Selection bias occurred, for example, as the majority of the persons in the studied population had a moderate to high educational level. This selection bias also appeared in the qualitative phase because of difficulties in reaching lower educated persons for taking part in the interviews. Consequently, results could not be generalized to all older workers. However, this study showed important findings which should be studied in a more generalizable population (or other subgroups) in future studies. Especially, given the expected increase demands for paid jobs by (low educated) persons who have reached the statutory retirement age. Second, the quantitative data relied on self-reported questionnaires. Underlying motivations might therefore be superseded and questions might be misinterpreted by respondents. Third, results derived from qualitative studies depend on the interpretation of the researchers; quotes relied on notes rather than verbatim text. To minimize this bias, the interviews were conducted by two independent persons. Analyzing data into themes took place independently and the results were compared in the final stage.

### Implications for research and practice

This study has a number of practical implications and provides directions for future research. First, personal characteristics, work characteristics and contextual factors include important determinants of working beyond retirement. This should be taken into account by policy makers as well as those developing interventions aimed at the enhancement of opportunities for working beyond retirement, for example, by taking a multifactorial approach. Second, to enable older workers to continue to work beyond the age of 65 some reluctant prejudices from the employers’ perspective need to be addressed and solved. As current research on the employers’ perspective is rare, further research into barriers and facilitators from a employers’ perspective is needed. Third, as working beyond retirement is a relatively new phenomenon, the consequences for health outcomes are still unknown. One could argue that the prolonged exposure to unfavorable work characteristics could have a negative impact on physical and mental health. However, as mentioned, earlier bridge employment offers the opportunity to improve quality of life through being physically, mentally and socially active. In both cases, individual differences are expected. A better understanding of the outcomes for various groups, for example, workers with different educational levels or for specific diseases, is needed to better tailor future jobs and policies regarding working beyond the retirement age.

## Concluding remarks

In line with the study by de Wind et al. (2016), we conclude that working beyond retirement is associated with good physical health, developmental proactivity, interesting work, appreciation, and voluntary work. Furthermore, this study showed that working beyond retirement—in a predominantly highly educated population—is mainly motivated by the desire to contribute to society. Additionally, the precondition of employers’ willingness to hire older workers and to provide them support and opportunities was highlighted. Therefore, personal characteristics as well as work characteristics and contextual factors seemed to be essential in the promotion to work beyond the statutory retirement age.

## What is new in the paper?

- The contribution of different determinants is studied using a mixed-methods design.
- Especially, physical health, interesting work and appreciation contributed strongly to working beyond retirement. Additionally, the employers’ willingness and the desire to contribute to society is highlighted.
- Enabling workers to work past the age of 65 years might be supported by work-related interventions promoting health, appreciation and reducing reluctant prejudices.

## Appendix A

See Table 4.

**Table 4:** The semi-structured interview guide used during the qualitative phase, subdivided for individuals working beyond retirement and retirees

	Working beyond retirement (*N* = 15)	Retired (*N* = 15)
Introduction		
1. Can you please introduce yourself?	✓	✓
Reasons for working beyond retirement/retirement		
2. You’re currently working beyond retirement, can you explain why?	✓	
3. You’re currently retired, can you explain why?		✓
4. To what extent did your health play a role in the decision to work beyond retirement/retire?	✓	✓
5. To what extent did your financial situation play a role in the decision to work beyond retirement/retire?	✓	✓
6. To what extent did your work environment play a role in the decision to work beyond retirement/retire?	✓	✓
Timing		
7. At what point in your career did you decide to continue working/retire?	✓	✓
8. Did you continue to work immediately after reaching the statutory retirement age?	✓	
Considerations and expectations		
9. Have you also considered to stop working and retire?	✓	
10. Have you considered to work beyond retirement?		✓
11. What changes have occurred in the job you are currently involved in versus the work you performed before you turned 65 years?	✓	
12. Can you explain your retirement expectations?		✓
13. To what extent does your retirement meet to these expectations?		✓
Persons who played a role in the decision making process		
14. You’ve decided to continue to work, can you tell me who played a role in this decision?	✓	
15. You’ve decided to retire, can you tell me who played a role in this decision?		✓
16. What do they think now? How did they react to your decision to continue/retire?	✓	✓
Planning for the future		
17. Until what age do you plan to continue?	✓	
18. What do you intend to do if you decide to stop working?	✓	
19. Have you considered doing this earlier?	✓	
20. Do you know persons working beyond retirement and can you imagine why they do so?		✓
21. What, to your opinion, could persons aged over 65 years of age encourage to work beyond retirement?	✓	✓
